# Secretion of a mammalian chondroitinase ABC aids glial integration at PNS/CNS boundaries

**DOI:** 10.1038/s41598-020-67526-0

**Published:** 2020-07-09

**Authors:** Philippa M. Warren, Melissa R. Andrews, Marc Smith, Katalin Bartus, Elizabeth J. Bradbury, Joost Verhaagen, James W. Fawcett, Jessica C. F. Kwok

**Affiliations:** 1 Department of Clinical Neurosciences, John Van Geest Centre for Brain Repair, University of Cambridge, Cambridge, CB2 0PY UK; 2 Wolfson Centre for Age Related Diseases, Institute of Psychiatry, Psychology and Neuroscience, King’s College London, Guy’s Campus, London Bridge, London, SE1 1UL UK; 3 Department of Physiology, Development and Neuroscience, University of Cambridge, Cambridge, CB2 0PY UK; 4 Faculty of Environmental and Life Sciences, University of Southampton, Southampton, SO17 1BJ UK; 5 Netherlands Institute for Neuroscience, Institute of the Royal Netherlands Academy of Arts and Sciences, Amsterdam, The Netherlands; 6 Centre for Reconstructive Neuroscience, Institute of Experimental Medicine, Czech Academy of Sciences, Videnska 1083, 14220 Prague 4, Czech Republic; 7 School of Biomedical Sciences, Faculty of Biological Sciences, University of Leeds, Leeds, LS2 9JT UK

**Keywords:** Extracellular matrix, Cellular neuroscience, Spinal cord injury

## Abstract

Schwann cell grafts support axonal growth following spinal cord injury, but a boundary forms between the implanted cells and host astrocytes. Axons are reluctant to exit the graft tissue in large part due to the surrounding inhibitory environment containing chondroitin sulphate proteoglycans (CSPGs). We use a lentiviral chondroitinase ABC, capable of being secreted from mammalian cells (mChABC), to examine the repercussions of CSPG digestion upon Schwann cell behaviour in vitro. We show that mChABC transduced Schwann cells robustly secrete substantial quantities of the enzyme causing large-scale CSPG digestion, facilitating the migration and adhesion of Schwann cells on inhibitory aggrecan and astrocytic substrates. Importantly, we show that secretion of the engineered enzyme can aid the intermingling of cells at the Schwann cell-astrocyte boundary, enabling growth of neurites over the putative graft/host interface. These data were echoed in vivo. This study demonstrates the profound effect of the enzyme on cellular motility, growth and migration. This provides a cellular mechanism for mChABC induced functional and behavioural recovery shown in in vivo studies. Importantly, we provide in vitro evidence that mChABC gene therapy is equally or more effective at producing these effects as a one-time application of commercially available ChABC.

## Introduction

Repair and recovery from spinal cord injury (SCI) is problematic due to the multitude of factors which need to be overcome including the large degree of cell loss, reduction in growth promoting molecules, and physical damage to axonal and neuronal pathways^1,2^. Principle amongst these are the upregulation of growth inhibitory chondroitin sulphate proteoglycans (CSPGs) present in the glial scar and extracellular matrix (ECM). The bacterial enzyme chondroitinase ABC (bChABC) has been used in vitro^3–6^ and in vivo^7–10^ to overcome this mechanical and chemical impediment. Through catabolism of glycosaminoglycan (GAG) side chains from the chondroitin sulphate (CS) backbone^11^, the enzyme digestion increases plasticity^9,12,13^, neuroprotection^14^, and facilitates functional recovery following SCI^7,15,16^.

Second-generation enzyme development has enabled long-term delivery of stabilized ChABC. Thermostabilised ChABC has yielded functional gains in locomotor activity and serotonergic sprouting when secreted from a scaffold system and used in combination with neurotrophic factors following thoracic dorsal over-hemisection^17^. Transgenic mice have been engineered to secrete ChABC from reactive astrocytes^18^ which upregulate at the site of CNS injury. Through site-directed mutagenesis of specific *N*-glycosylation sites, we have designed a version of the bChABC enzyme that can be secreted from mammalian cells^19^ (mChABC). This has been incorporated into lentiviral vectors and shown to be active following in vivo transduction^20–23^. The work of Kanno et al*.*^24^ showed that mChABC was able to digest CS-GAGs within Schwann cell grafts, aiding the integration of this tissue with host cells. However, the cellular mechanisms governing these effects have not been determined. Here we assessed this process.

Specifically, we examine the efficacy of the mChABC over bChABC through in vitro models of Schwann cell migration, adhesion, and glial confrontation. Transplantation of Schwann cells holds considerable promise as a therapy for SCI^25–28^. However, the formation of sharp boundaries between the Schwann cells and astrocytes act as a barrier to regeneration^29,30^. Nonetheless, if the host and implanted cells can intermingle, axonal growth over the two tissue types may be facilitated^31,32^. Assessment of the intermingling of cells within our functional assays reveals potential integration of cells within astrocytic cellular populations. We demonstrate that high quantities of active and stable mChABC can be secreted from Schwann cells. Additionally, the activity of this enzyme can facilitate in vitro Schwann cell migration, adhesion, and intermingling within inhibitory astrocytic environments. The mChABC mediated change in cellular activity enables significant increases in the number of growing neurites to cross the PNS/CNS interface. In vivo, mChABC enabled Schwann cell intermingling with astrocytes at lesion borders*.* The identified process through which mChABC affects cellular activity explains the behavioural and regenerative effects of the enzyme in previous in vivo studies. Furthermore, we demonstrate that our engineered mChABC enzyme produces effects equivalent to, or greater than, the commercially available bChABC.

## Results

### Expression, secretion, and stability of mChABC from transduced Schwann cells

**Figure 1:**
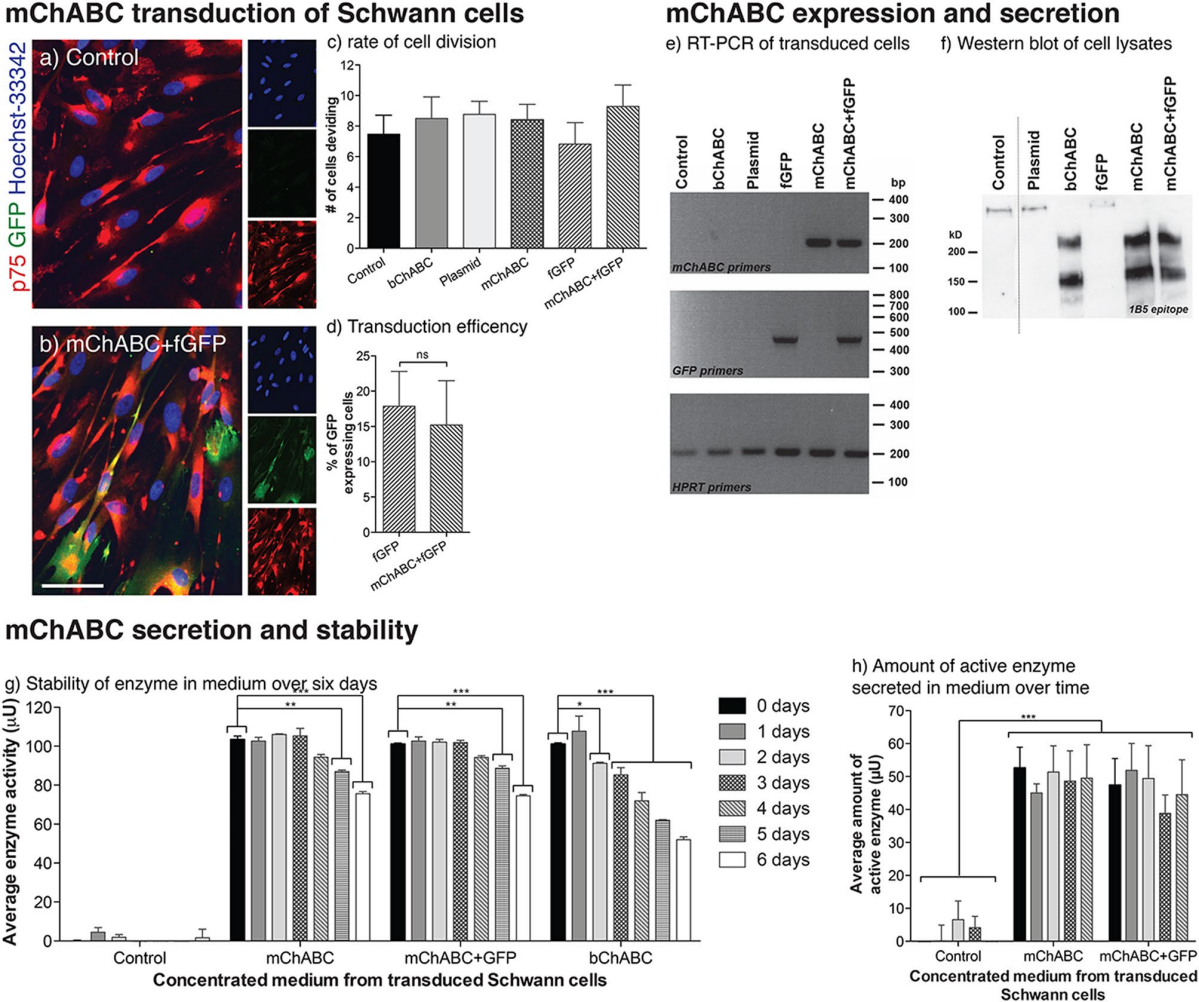
mChABC can be transduced, expressed, and secreted by Schwann cells. Schwann cells were control, bChABC treated, or transduced with LV-plasmid control, LV-mChABC, LV-fGFP, or LV-mChABC + LV-fGFP (**a**–**d**) Images show (**a**) LV-plasmid control and (**b**) LV-mChABC + LV-fGFP transduced cells immunostained for Hoechst-33342 (blue); GFP (green) and p75 (red), scale bar = 40 μm. (**c**) Transduction did not alter rate of Schwann cell division (N = 4, one-way ANOVA F(5,18) = 0.528, *p* = 0.753). (**s**) The same transduction efficiencies were achieved for LV-fGFP and LV-mChABC + LV-fGFP cells (N = 30, one-way ANOVA F(5,174) = 6.932, *p* < 0.0001, post hoc test p = ns). (**e**–**f**) mChABC is expressed and secreted by transduced Schwann cells (for full gel see Supplementary Fig. 2). (**e**) RT-PCR of cells with HPRT, mChABC and GFP primers. (f) Western blot of cell medium probed using anti-1B5 antibody. Dashed line denotes area of cropped image (see Supplementary Fig. 2). DNA and proteins were quantified to ensure equal gel loading. (**g**–**h**) Transduced Schwann cells secrete constant amounts of stable mChABC. (**g**) 100μU of secreted mChABC is more stable at 37 °C than 100μU of bChABC (N = 3, two-way ANOVA: days post transduction F(6,84) = 48.23, *p* < 0.0001, transduced cell populations F(5,84) = 219.92, *p* < 0.0001). (**h**) Amount of active mChABC secreted by transduced Schwann cells over 4 days (N = 3, two-way ANOVA: days post transduction F(6,50) = 0.32, *p* = 0.8625, cells transduced F(4,50) = 66.01, *p* < 0.0001).

In order to assess the effect that a mammalian cell-secreted ChABC has on cellular migration and adhesion, the mChABC construct must be delivered into specific cells, expressed, and produced in an active and stable form. Primary Schwann cells were transduced with either LV-mChABC or LV-fGFP or co-transduced with both vectors (Fig. 1a–d). Following immunostaining for the nuclear protein Ki67 (illustrative of cellular interphase), the transduction procedure was shown not to alter the proliferation rate of cells, despite the use of polybrene (Fig. 1c)^33^. Co-transduction of LV vectors using the same viral backbone and under the same promoter have been shown to have similar transduction efficiencies^34–37^ (despite differences in the size of RNA packaged). Thus, GFP positive cells were determined indicative of transduction efficiency for all cell populations. Utilising LV-mChABC and LV-fGFP, both under the CMV promotor and at MOIs given above, a transduction efficiency of ~ 15% was determined in cellular populations of 100% p75 positive Schwann cells (Fig. 1a,b,d). This was not significantly different from the transduction of LV-fGFP alone (*p* = ns). RT-PCR confirmed expression of mChABC and fGFP specifically in the transduced cellular populations (Fig. 1e).

Concentrated medium collected over 24 h from the transduced and control Schwan cell populations (at 48–62 h following transduction) were assayed by Western blot to assess secretion and activity of mChABC (Fig. 1f). Probed with anti-1B5, blots exhibited banding at ~ 150 and 210kD in both the mChABC transduced populations and the bChABC treated control. These data illustrate total CS-GAG removal from medium-soluble CSPGs due to the presence of active ChABC. The activity of the secreted enzyme was further explored using the CPC turbidity assay (Fig. 1g–h). Initially, medium from transduced Schwann cells was collected every 24 h for 4 days and then activity assayed. Data showed a population of 3 × 10^5^ Schwann cells (transduced at a rate of ~ 15%) consistently and reliably yielded ~ 50 μU of active mChABC over a 24-h period (equals ~ 0.16 mU of active ChABC/150 cells transduced with the virus; Fig. 1h). Further, 100 μU of active mChABC from these cells was incubated at 37 °C for 6 days. The secreted mChABC was shown to have superior thermostability in culture medium compared to commercially available bChABC remaining at a plateau of activity for 5 rather than 3 days and exhibiting less loss of total activity over time (Fig. 1g). It is likely that had we measured enzyme activity at earlier time points it would have been initially higher than these data suggest due to the half-life of the enzyme at 37°C^19,38^. Nonetheless, these data demonstrate that Schwann cells can be reliably transduced with LV-mChABC. Further, these results show that the biologically active enzyme is expressed and secreted from these cells in high yield and with stability. These cells were used in all subsequent functional assays to determine the effect of mChABC upon Schwann cell migration and adhesion.

### mChABC modulates Schwann cell migration, adhesion and integrin signalling

Using the inverted coverslip migration assay, we confirmed previous findings, showing that Schwann cell migration and adhesion is dependent upon the substrate (Supplementary Fig. 1a–e)^39–42^. The number and distance of migration was reduced (~ 20%) when grown on an inhibitory CSPG containing substrate as compared to laminin (Supplementary Fig. 1d). The aggrecan substrate was selected as an inhibitor of Schwann cell migration on astrocytes which is mediated through glial-cell produced aggrecan^41^. Interestingly, combining the laminin and aggrecan substrates led to a ~ 50% reduction in cell number and distance of migration compared to laminin alone. This suggests that the inhibitory effect induced by aggrecan on cellular migration can be partially overcome through the presence of positive migratory cues (Supplementary Fig. 1a–e). These data were similar to the effect of substrate specific binding upon Schwann cell adhesion (Supplementary Fig. 1h–j). Adhesion was inhibited on the aggrecan substrate, but significantly increased when conducted in the presence of laminin. This substrate specific effect was independent of cell division (Supplementary Fig. 1f–g). The number and distance of Schwann cell migration was not different when cell division was inhibited with aphidicolin. These data demonstrate that experimental differences are substrate dependent.

**Figure 2:**
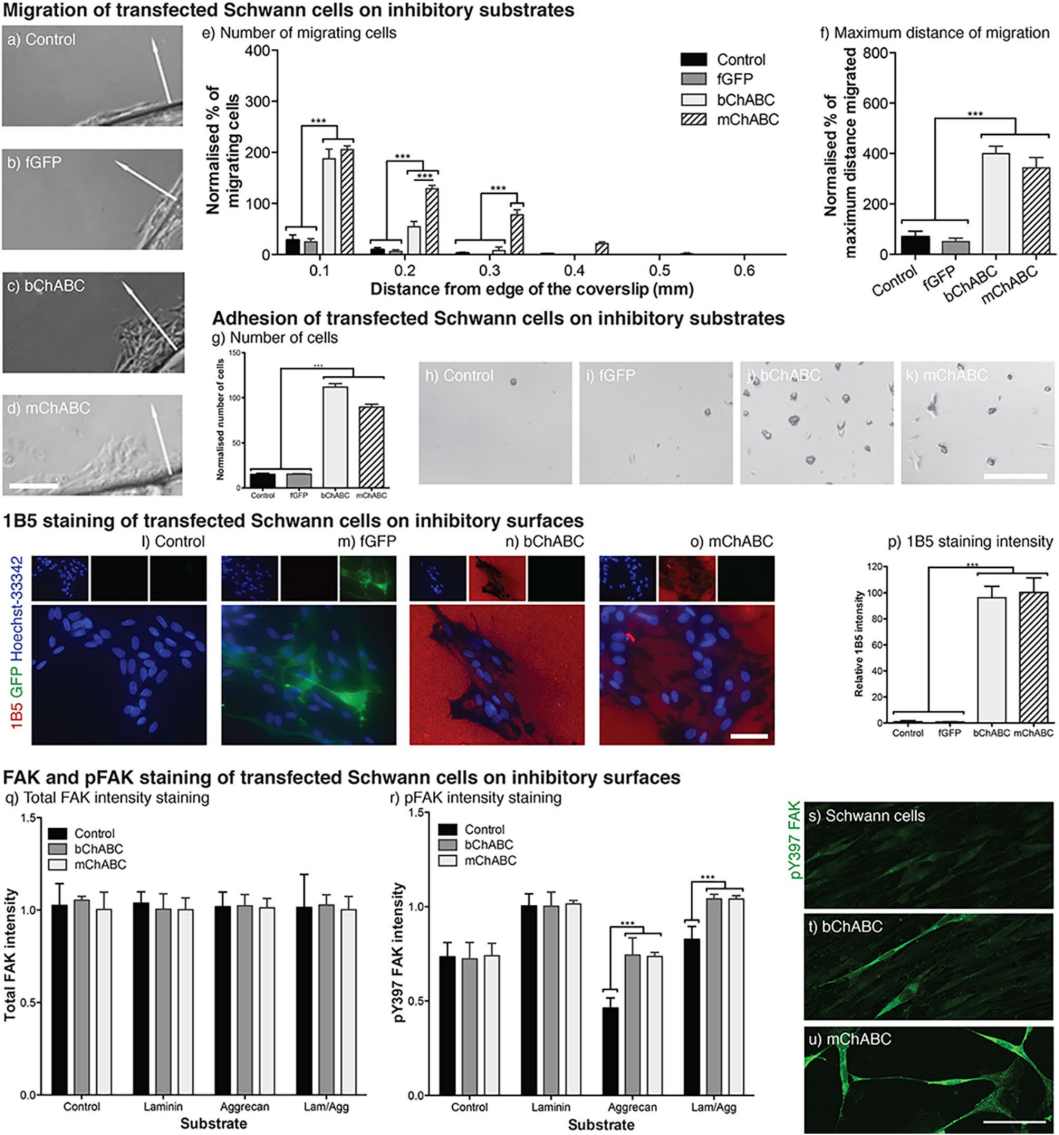
mChABC increases Schwann cell migration and adhesion on aggrecan by removal of CS-GAGs. (**a**–**f**) Migration of Schwann cells on aggrecan showing (**a**) control, (**b**) LV-fGFP, (**c**) bChABC treated, and (d) LV-mChABC populations. Scale bar = 100 μm, arrows indicate direction of migration. Quantification of (e) cells migrating (N = 5, two-way ANOVA: cell type F(3,84) = 131.34, *p* < 0.0001, distance F(5,84) = 160.84, *p* < 0.0001) and (**f**) maximum distance (N = 5, one-way ANOVA: F(3,14) = 39.53, *p* < 0.0001). (**g**–**k**) mChABC increases cellular adhesion on aggrecan (**g**) to levels comparable with bChABC (N = 3, one-way ANOVA: F(3,12) = 68.61, *p* < 0.0001). Images showing (**h**) control, (**i**) LV-fGFP, (**j**) bChABC treated, and (**k**) LV-mChABC transduced cells. Scale bar = 100 μm. (**l**–**p**) anti-1B5 staining (red) of Schwann cells on aggrecan showing (**l**) control, (**m**) LV-fGFP, (**n**) bChABC treated, and (**o**) LV-mChABC populations immunostaned for anti-GFP (green) and Hoechst-33342 (blue) quantified in p) (N = 20, one-way ANOVA F(3,76) = 126.8, *p* < 0.0001). Scale bar = 40 μm. (**q**) Total FAK (N = 3, two-way ANOVA: cell type F(3,224) = 1.15, *p* = 0.329, substrate F(3,224) = 0.26, *p* = 0.8537) and (**r**) pFAK (N = 3, two-way ANOVA: cell type F(3,224) = 69.06, *p* < 0.0001, substrate F(3,224) = 491.82, *p* < 0.0001) levels in transduced cells on different substrates. Images show anti-pY397 FAK (green) staining of (**s**) control, (**t**) bChABC treated and (**u**) LV-mChABC transduced Schwann cells on an aggrecan substrate. Scale bar = 100 μm.

To assess the effect mChABC has upon Schwann cell migration over inhibitory substrates, we performed the inverted coverslip migration assay using the transduced cell populations. When placed upon an inhibitory aggrecan substrate, naïve and fGFP transduced cells typically showed very little migration (~ 25%) and adhesion (~ 15%) of cells. However, mChABC secretion from cells enabled greater numbers of cells to migrate (~ 200%) a longer distance (~ 400%) than the control populations (Fig. 2a-d). Indeed, these cells migrated the same distance as those on the aggrecan substrate pre-treated with bChABC (Fig. 2f). Interestingly, a greater number of cells were found to migrate larger distances with mChABC than with bChABC (Fig. 2e). The increased number and distance of mChABC-expressing cellular migration compared to those on a bChABC-treated substrate was likely due to continuing modification of the ECM throughout the time of the assay. Similar effects were seen with cellular adhesion (Fig. 2g–k). The secretion of mChABC from Schwann cells enabled greater numbers of cells to adhere to the aggrecan substrate. These data were non-divergent from substrate which was pre-treated with bChABC (*p* > 0.05). These effects were likely caused by the removal of CS-GAG chains from the aggrecan substrate (Fig. 2l–p). Both mChABC and bChABC were shown to expose the 1B5 stub, indicative of complete CS-GAG removal from the aggrecan substrate, with equal efficiency (Fig. 2n-p; *p* > 0.05).

To assess the mechanism through which mChABC removal of CS-GAGs may facilitate Schwann cell migration, we assessed integrin signalling. CSPGs have been shown to interfere with aggrecan and laminin binding to cells^31,43–47^. When active, integrins bind with high-affinity to their specific ligands, which can facilitate neurite outgrowth^48,49^. Further, Schwann cell migration has been shown to be integrin dependent^41^. To assess the effect of mChABC treatment upon this signalling pathway, we determined the level of naïve and transduced Schwann cell total FAK and phospho-FAK (pFAK) of the tyrosine 397 residue when grown on different substrates (Fig. 2q–u). The tyrosine 397 residue on FAK is the first phosphorylation site after integrin activation. Its state of phosphorylation alters depending upon the substrate upon which cells are seeded^41,44,50^. Regardless of the cell type used or substrate upon which cells were grown, levels of total FAK (phosphorylated and non-phosphorylated) in all cells remained constant (Fig. 2q). Further, all cells showed increases in pFAK when grown on laminin (Fig. 2r) and naïve Schwann cells showed decreases in pFAK staining when grown on aggrecan and laminin/aggrecan substrates. However, cells secreting active mChABC showed significantly increased pFAK staining when grown on aggrecan or laminin/aggrecan compared to untransduced Schwann cells (Fig. 2r-u). The effect of mChABC was not significantly different from that caused by treatment of the cells with bChABC (*p* = 0.05). These data suggest that ChABC treatment mediates its effect upon Schwann cell migration partly through an integrin dependent pathway due to CS-GAG removal.

### mChABC increases Schwann cell migration and adhesion on astrocytes

**Figure 3:**
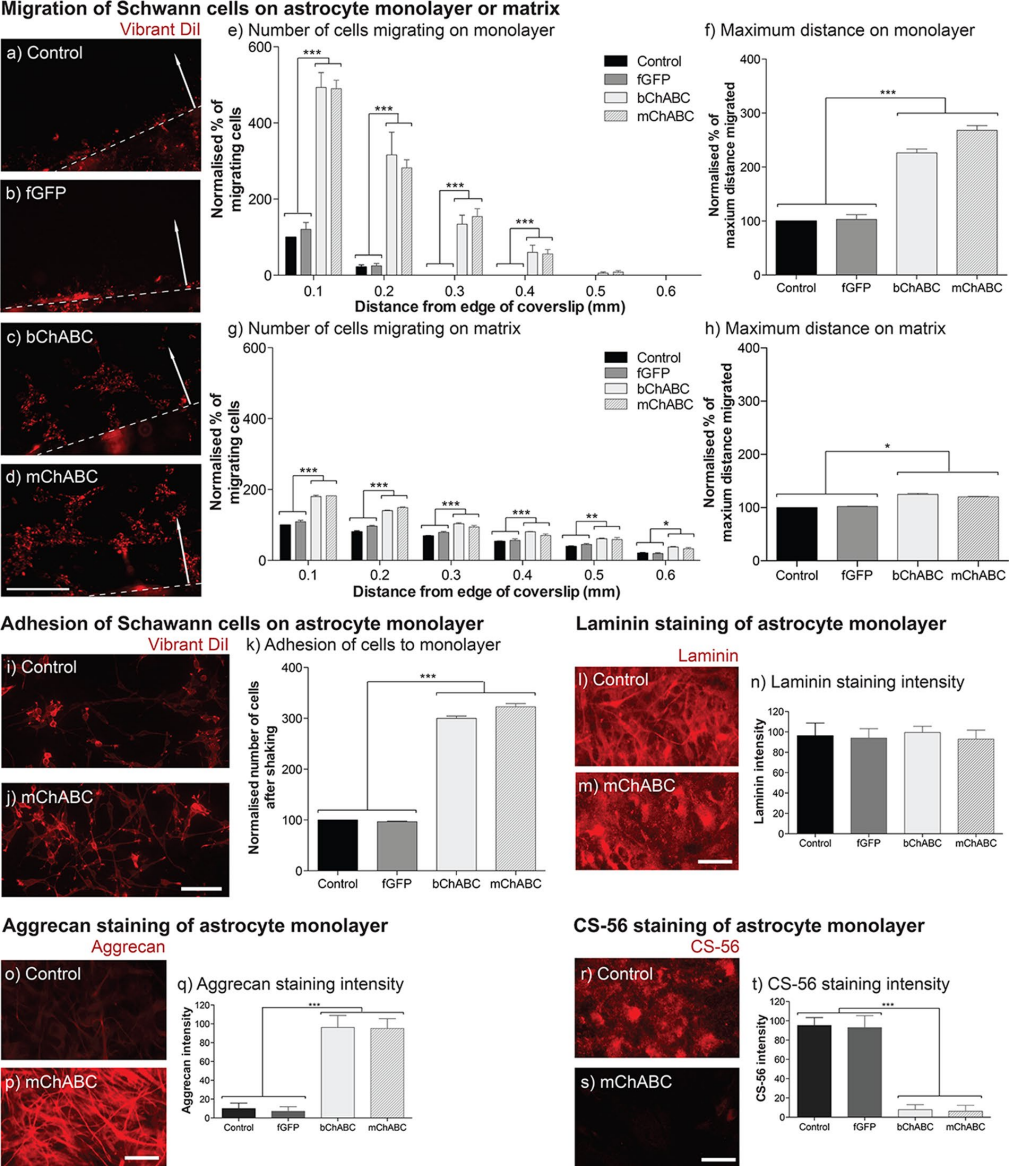
mChABC increases Schwann cell migration and adhesion on astrocytes through CS-GAG removal. (**a**–**f**) Migration of Schwann cells (red; vibrant DiI) on astrocytes showing (**a**) control, (**b**) LV-fGFP, (**c**) bChABC treated, and (**d**) LV-mChABC populations. Scale bar = 100 μm; arrows indicate direction of migration; dashed line, the coverslip edge. Quantification of (**e**) cells migrating (N = 5, two-way ANOVA: cell type F(3,150) = 133.78, *p* < 0.0001, distance F(5,150) = 177.42, *p* < 0.0001) and (**f**) maximum distance (N = 5, one-way ANOVA: F(3,25) = 138.3, *p* = 0.0203). Cells were migrated on astrocyte matrix showing g) cells migrating (N = 5, two-way ANOVA: cell type F(3,48) = 311.15, *p* < 0.0001, distance F(5,48) = 897.39, *p* < 0.0001) and (**h**) maximum distance (N = 5, one-way ANOVA: F(3,8) = 134.4, *p* < 0.0001). (**i**–**k**) mChABC increases cellular adhesion on astrocytes. Showing (**i**) control, and (**j**) LV-mChABC populations stained with p75 (red) with (**k**) quantification (N = 20, one-way ANOVA: F(3,12) = 67.75, *p* < 0.0001). Scale bar = 100 μm. (**l**–**m**) anti-laminin, (**o**–**q**) anti-aggrecan (Cat 301), and (**r**–**t**) anti-CS-56 staining of astrocyte monolayer treated with medium from control, bChABC treated, LV-fGFP, and LV-mChABC populations. Scale bar = 40 μm. Quantified in (**k**) for laminin (N = 20, one-way ANOVA: F(3,76) = 1.873, *p* = 1.412); (**n**) aggrecan (N = 20, one-way ANOVA: F(3,76) = 614.6, *p* < 0.0001) and (**t**) CS-56 (N = 20, one-way ANOVA: F(3,76) = 708.6, *p* < 0.0001).

With the success of mChABC to enable Schwann cell migration and adhesion on pure aggrecan, we next determined if similar results could be generated when Schwann cells were seeded onto an astrocytic substrate. We confirmed that naïve and fGFP-expressing Schwann cells show little migration and adhesion on astrocytic monolayers due to inhibitory molecules secreted by these cells (Fig. 3a–k). However, secretion of mChABC caused significant increases in the number of adherent cells and maximum distance of cellular migration (~ 500%, Fig. 3a–f). These data were not divergent from that obtained following pre-treatment of the astrocytic monolayer with bChABC (*p* = ns). Interestingly, performing a similar experiment on the astrocytic matrix produced a similar trend (Fig. 3g–h). However, numbers of cells and maximum distance migrated were fewer once normalised to control cell (naïve) populations, although, more cells were able to travel, on average, a greater distance upon the matrix. Schwann cell migration is mediated through a balance of inhibitory and permissive factors. Most CSPGs produced by astrocytes are secreted and not retained on the cell surface^6,51,52^. These data suggest that the matrix produced by astrocytes alone contains components inhibitory to Schwann cell migration and adhesion. However, there are a greater number of positive cues retained on the surface of the glial cells which are exposed following CS-GAG removal which can aid cellular migration. Indeed, immunostaining revealed that while neither bChABC pre-treatment nor mChABC activity diminished laminin staining on the astrocytic monolayer (Fig. 3l–n), the activity of both forms of the enzyme acted to expose the aggrecan protein core bound to the cell surface shown through anti-aggrecan staining (Fig. 3o–q). Furthermore, this increase in staining associated with significant removal of CS-GAGs were confirmed by a reduction in CS-56 staining (Fig. 3r–t). The removal of CS-GAGs from the surface of the cellular monolayer, through the action of either mChABC or bChABC, was shown to cause an increase in the adhesion of Schwann cells to astrocytes (~ 340%, Fig. 3i–k). Together, these data show mChABC can digest inhibitory CS-GAGs from the astrocyte surface and ECM with the equivalent efficiency to commercial bChABC. The breakdown of these sugar chains facilitates Schwann cell migration and adhesion on astrocytes, two factors important in cellular boundary integration.

### mChABC increases Schwann cell intermingling of cells at astrocyte boundaries

**Figure 4:**
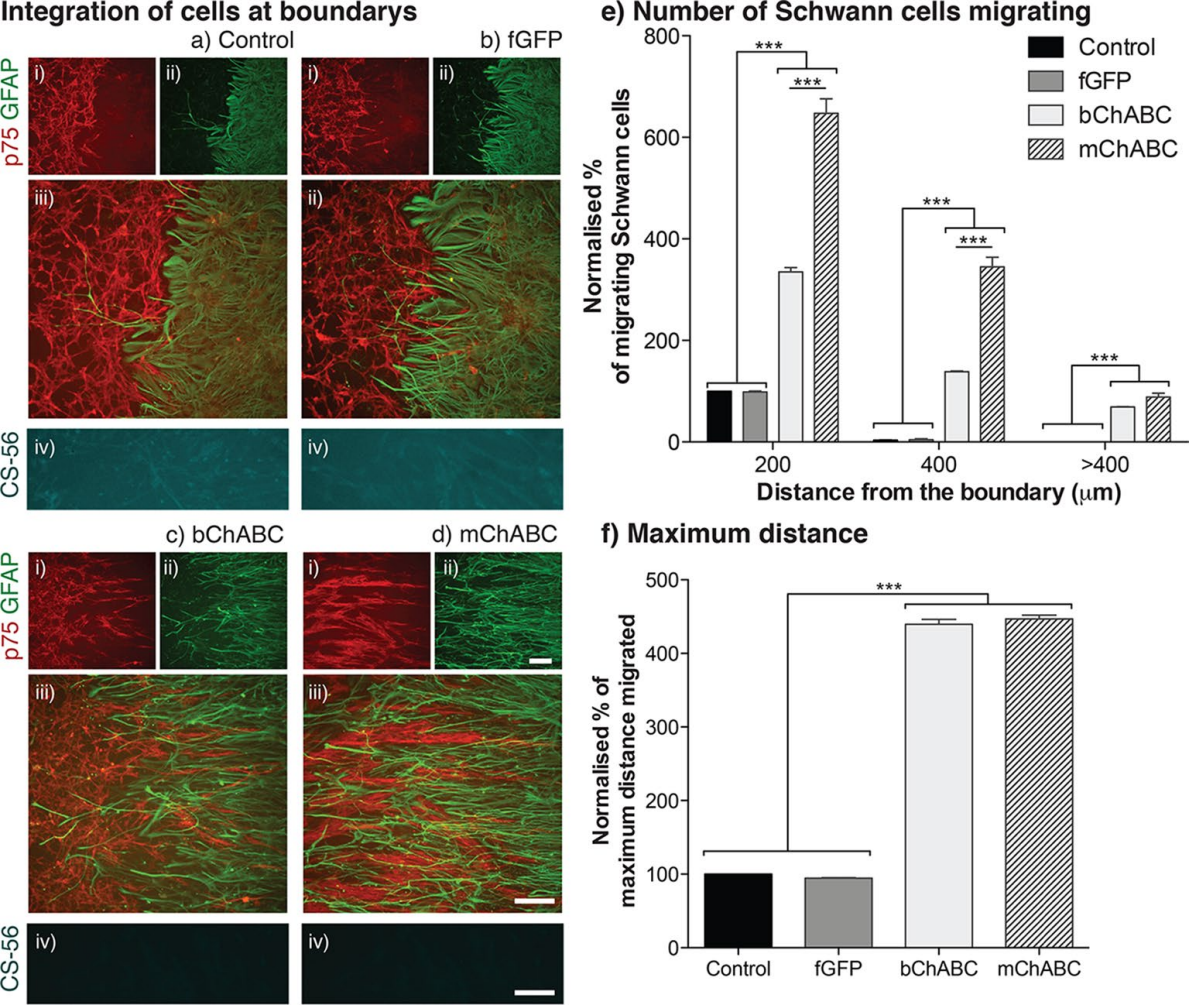
mChABC enables cellular intermingling at the Schwann cell astrocyte boundary. (**a**–**d**) Schwann cells labelled for anti-p75 [red; (i) and astrocytes for anti-GFAP (green; ii) for (**a**) control, (**b**) LV-fGFP, (**c**) bChABC treated, and (**d**) LV-mChABC populations where iv] shows immunostaining against anti-CS-56 (cyan). Scale bar = 100 μm. (**e**) mChABC secretion increases Schwann cell-astrocyte intermingling (N = 4 two-way ANOVA: cell type F(3,36) = 655.41, *p* < 0.0001, distance F(2,36) = 623.08, *p* < 0.0001). (**f**) mChABC increases maximum distance of Schwann cell migration at the boundary (N = 5, one-way ANOVA: F(3,12) = 212.3, *p* < 0.0001).

In order to assess the effect mChABC has upon cellular intermingling, we performed the Schwann cell-astrocyte confrontation assay (Fig. 4a-d). This closely models the barrier which forms between the two glial cell types following tissue transplantation^29,39,42,53^. We plated dense populations of naïve or transduced Schwann cells together with astrocytes 0.5 mm apart on a PDL and laminin coated slide. Using an established protocol^53^, the cell populations requiring bChABC treatment were exposed to 100 mU of the enzyme for 1 h daily while the boundary formed. Fresh medium was placed on all assays, regardless of treatment group, every 24 h. After 5 days, the cell populations had grown together, forming a distinct straight boundary with only a few Schwann cells penetrating the astrocyte population (Fig. 4a).

All treatment groups exhibited more intense GFAP immunoreactivity at points of contact with anti-p75 positive Schwann cells. This suggests that neither mChABC nor bChABC caused a change in the astrocytic reaction to Schwann cell contact^54^. Nonetheless, secretion of the enzyme did affect cell behaviour. mChABC secretion from Schwann cells produced an increase in the number of Schwann cells intermingling with the astrocytes (~ 640%, Fig. 4b) and the maximum distance of migration into the astrocyte population (Fig. 4e-f). This was most evident up to 400 μm from the edge of the boundary where mChABC was shown to facilitate a greater number of cells penetrating through the glial interface (*p* < 0.0001). Both mChABC and bChABC caused a similar reduction in CS-GAGs over both the Schwann cell and astrocyte (*p* = ns) populations shown through a reduction in CS-56 staining (Fig. 4a-d). These data suggest that secretion of mChABC from Schwann cells enables CS-GAG removal that can facilitate the penetration of Schwann cells into typically inhibitory astrocytic populations.

### mChABC increases neuronal outgrowth over PNS/CNS interfaces

**Figure 5:**
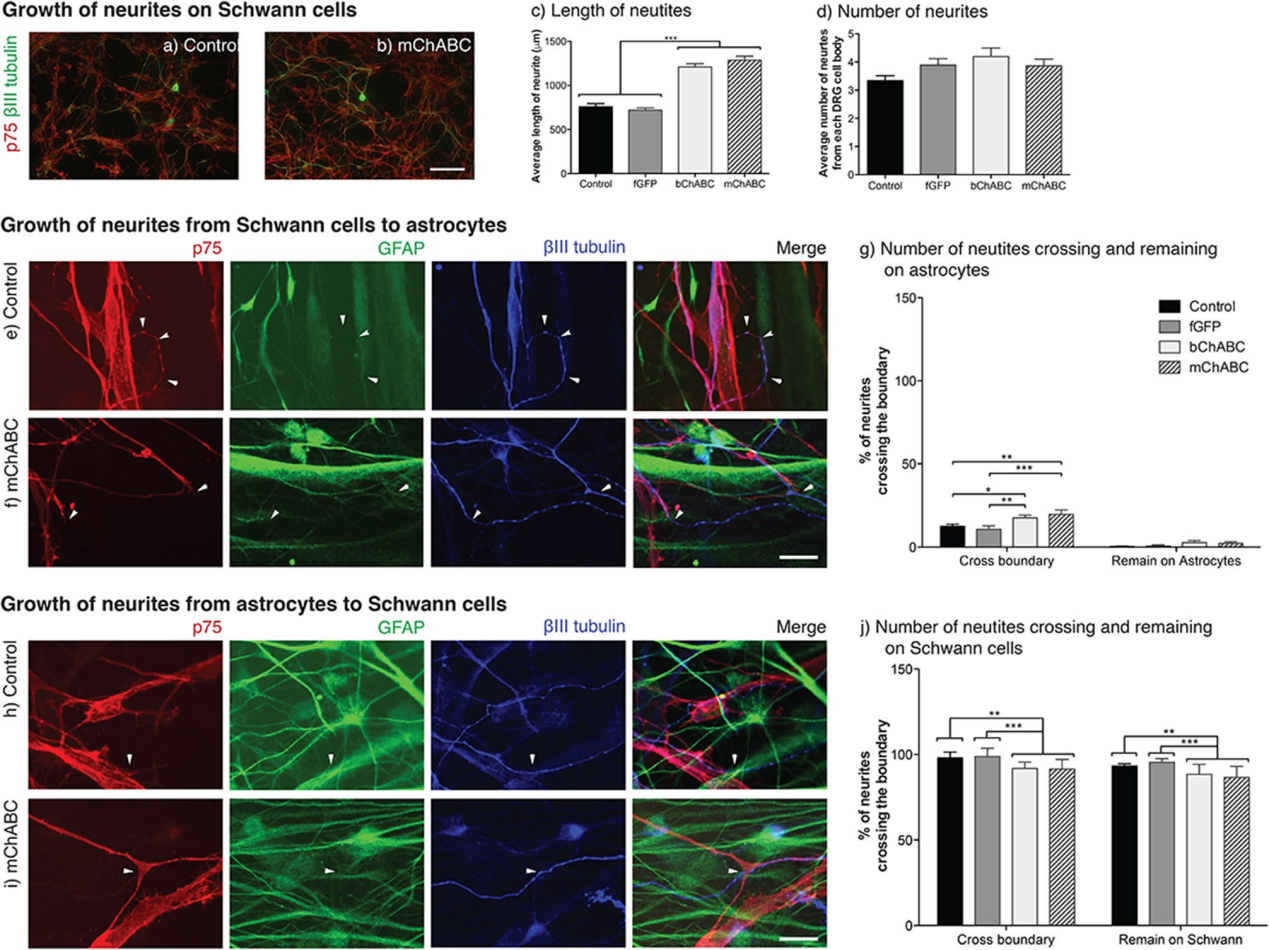
mChABC secretion aids neurite outgrowth over the Schwann cell-astrocyte boundary. (**a**–**b**) Images show Schwann cells (anti-p75; red) and DRG neurites (anti-βIII-tubulin; green) for (**a**) control, and (**b**) mChABC transduced cells. mChABC increases (**c**) the length of DRG neurites growing on Schwann cells (N = 3, one-way ANOVA: F(3,172) = 62.25, *p* < 0.0001) but (**d**) not the number of neurites (N = 3, one-way ANOVA: F(3,172) = 2.085, *p* = 0.1039). (**e**–**g**) Growth of neurites from (**e**) control, and (**f**) mChABC transduced Schwann cells (anti-p75; red) to astrocytes (anti-GFAP; green) or (**h**–**j**) astrocytes to Schwann cells with DRG neurites (anti-βIII-tubulin; blue). (**g**) mChABC increases the number of neurites crossing from Schwann cells to astrocytes (N = 3, one-way ANOVA: F(3,7) = 4.741, *p* = 0.0413). However, it does not increase the number that remain on the glial cell (N = 3, one-way ANOVA: F(3,7) = 2.07, *p* = 0.1928). (**j**) mChABC increases the number of neurites crossing from Schwann cells to astrocytes (N = 3, one-way ANOVA: F(3,7) = 0.8132, *p* = 0.05259). However, it does not increase the number that remain on the glial cell (N = 3, one-way ANOVA: F(3,7) = 3.804, *p* = 0.1148). For all panels, scale bar = 100 μm.

The increased cellular intermingling at the Schwann cell astrocyte boundary caused by secretion of mChABC illustrates that through CS-GAG breakdown, the enzyme can overcome the inhibitory effects of CSPGs. In addition to their effects on cell migration, high concentrations of CSPGs cause growth cones to enter a dystrophic state which, while dynamic^55^, prevents axonal or neuronal growth^6,56–59^. To assess whether mChABC could mediate a return of neurite outgrowth due to CS-GAG digestion, dissociated DRGs were seeded upon confluent monolayers of naïve, mChABC-transduced, or bChABC-treated Schwann cells and left to grow for 48 h (Fig. 5a-d). DRG neurite growth was closely associated with the long processes of Schwann cells in all treatment conditions. No significant difference in DRG morphology or the number of neurites produced by each cell body was noted between treatment groups (Fig. 5a-d). However, secretion of mChABC from Schwann cells did increase the length of the DRG neurites (~ 500 μm increase; Fig. 5b-c). This was not significantly different from the effect caused by bChABC treatment of the Schwann cell monolayer (*p* = ns). These data confirm that, even on a substrate typically permissive to neurite outgrowth, CS-GAG breakdown can further facilitate the growth of sensory axons. Interestingly, secretion of mChABC from Schwann cells did not alter the myelination of neurites grown in culture (Supplementary Fig. 3). This would suggest that the properties of Schwann cells potentially beneficial to functional recovery following spinal cord injury have not altered despite mChABC transduction.

Next, we determined if the mChABC mediated intermingling of Schwann cell and astrocytes would enable primary sensory neurons to grow over the glial cell interface (Fig. 5e–j). We seeded ~ 100 dissociated DRG neurons over the established Schwann cell-astrocyte boundary. Schwann cells were either naïve, transduced, or treated with bChABC. The DRGs were plated close to the boundary so that growing axons could have access to both cell types. Following DRG seeding, control cultures showed no increase in p75 positive cells in astrocytic territory, indicating that satellite p75 + cells did not migrate from the DRG cell body onto the astrocyte layer^53^. Away from the Schwann cell-astrocyte boundary, neurites grew readily on either cell type, albeit typically longer on Schwann cells. However, it was the behaviour of the neurites when forced to choose whether to cross from one cell-type to another at the Schwann cell- astrocyte interface that was interesting. When DRGs were initially plated upon naïve or fGFP expressing Schwann cells, the DRG neurites typically remained growing on this cell type with only ~ 11–13% of axons crossing to astrocytes (Fig. 5e, g). Further, less than 1% continued to grow on astrocytes for distances greater than 100 μm. Indeed, DRG neurites grew along Schwann cell processes, turning tight curves to avoid growth on astrocytic surfaces (Fig. 5f). However, in cultures where mChABC was secreted, a significantly greater number of DRG neurites crossed from Schwann cells to astrocytes (~ 17–19%; Fig. 5f–g). Further, more of these axons grew upon the astrocytic surface (*p* > 0.05). The effect mediated by the secretion of mChABC on DRG growth over the Schwann cell-astrocyte boundary was not significantly different from cultures treated with bChABC (Fig. 5g). In contrast to these data, the ability of DRG neurites to cross from astrocytes to Schwann cells was substantial (Fig. 5h-j). In cultures with naïve or fGFP expressing Schwann cells, ~ 95–98% of DRG neurites crossed from astrocytes to Schwann cells, with ~ 94–96% remaining on the latter population for over 100 μm (Fig. 5h, j). However, in cultures treated with either mChABC secretion or bChABC application there was a reduction in the percentage of neurites crossing from astrocytes to Schwann cells (~ 92- 94%; Fig. 5i-j). Further, slightly fewer (~ 89–91%) neurites remained growing upon the Schwann cells. These data suggest that the removal of CS-GAGs together with the deep penetration of cells through the Schwann cell-astrocyte boundary can cause a modest, yet significant increase in the number of axons that grow over the Schwann-cell astrocyte boundary. Further, this treatment modestly facilitates the growth of DRG neurites on typically less permissive astrocytic cells. The confrontation assay represents an in vitro model of axonal behaviour at the dorsal root entry zone (DREZ) and the graft-host interface following Schwann cell transplantation into an injured spinal cord.

### mChABC facilitates in vivo Schwann cell intermingling

To assess the degree to which intraspinal LV delivery of mChABC could facilitate neuronal growth and Schwann cell migration following experimental SCI compared to intraspinal injection of bChABC, we performed a thoracic contusion in adult rats (Supplementary Fig. 4). Two weeks following injury, LV-fGFP injected animals showed abundant CS-GAG chains around the lesion epicentre (Supplementary Fig. 4a). Substantially lower levels of these inhibitory molecules where shown following bChABC and LV-mChABC application (Supplementary Fig. 4b-c). These data demonstrate that LV-mChABC may be successfully transduced into adult cells in vivo and secreted in an active form which operates effectively. Further, the removal of CS-GAGs extended over large distances from the lesion site, indicative of both the spread of the enzyme in the tissue and the area of viral transduction (shown through fGFP; Supplementary Fig. 4d). Application of bChABC was shown to reduce the size of the lesion cavity following SCI as compared to controls (Supplementary Fig. 4d-e). This effect was augmented in mChABC animals who displayed not only a reduction in cavitation after SCI but also substantial axonal staining through the lesion site itself signifying a reduction in neuronal loss and potential sprouting (Supplementary Fig. 4f, e^i^-f^i^). These observations support our in vitro data, demonstrating that mChABC can facilitate neuronal growth within and across inhibitory environments and has the potential to exhibit superior effects to the bacterial enzyme.

Finally, we assessed the migration of Schwann cells into the astrocyte rich lesion penumbra from the periphery following SCI. Control animals showed no evidence of Schwann cell migration or intermingling around the lesion border and penumbra (Supplementary Fig. 4g). After administration of bChABC, a small number of Schwann cells were observed in this region (Supplementary Fig. 4h). However, in mChABC transduced animals a substantial number of Schwann cells were shown to intermingle with astrocytes in and around the lesion penumbra (Supplementary Fig. 4g). This study confirms our in vitro data, demonstrating that mChABC can facilitate the intermingling and growth of cells and has a potentially more profound effect than a one-time dose of the bacterial enzyme. Further, this demonstrates that mChABC can yield similar effects on adult cells in vivo as those demonstrated by postnatal animals in vitro.

## Discussion

Here we demonstrate that Schwann cells transduced with lentiviral vectors encoding ChABC can constitutively secrete high quantities of active mChABC. The activity of this second-generation enzyme causes profound CS-GAG removal in vitro, functionally altering Schwann cell migration, adhesion, and intermingling within inhibitory astrocytic environments whilst enabling neurite outgrowth over the PNS/CNS cellular interface. The mechanisms of mChABC on cellular activity explain the behavioural and regenerative effects of the enzyme in biochemical and in vivo studies. Importantly, we demonstrate that the effects produced from our modified mChABC enzyme are equivalent to, or greater than, the commercially available bChABC.

The functional expression and effects of mChABC secretion on the PNS/CNS boundary has not previously been shown. The mChABC mediated removal of inhibitory CS-GAGs from aggrecan, or astrocytic monolayers^41,42,53^ increased Schwann cell motility. Through GAG digestion, mChABC facilitated cellular-substrate attachment, resulting in increased numbers and distance of cellular migration^60^. Further, and superior to bChABC^61–63^, the action of mChABC facilitated the intermingling of Schwann cells with astrocytes at points of confrontation. Moreover, unlike bChABC^53^, mChABC additionally enabled astrocytes to migrate into a Schwann cell population. This would suggest that the continuous, global activity of mChABC is required to facilitate the migration of CNS glia, perhaps due to modifications to the ECM or cell-to-cell contact. This is echoed in populations of adult cells shown in our in vivo observations where migration and adhesion is prevented in part due to inhibitory CS-GAGs and inactivation of integrins^64–68^. As mChABC can influence these factors regardless of time following development or injury, our data support a universal process of mChABC-mediated GAG removal to aid the intermingling and growth of cells. Within our assay, the action of mChABC facilitated neuronal growth through CS-GAG removal and, potentially, by increasing neuroprotection^14^. The reduced inhibition and border irregularity enabled neurites to cross the PNS/CNS cellular boundary. This mechanism of mChABC action explains the similar in vitro and in vivo^29,53,69^ data and was supported by observations collected from our own in vivo work.

mChABC gene therapy following cervical and thoracic contusion injury has been shown to remove CS-GAGs, increasing neuroprotection through modulation of macrophage phenotype, and facilitate significant locomotor and sensorimotor function as demonstrated through ladder walking, axonal conduction, and grip strength^21,22^. Recently, it has been shown that use of an inducible mChABC vector facilitated sensory conduction and gross movement following short-term treatment, however, long-term treatment was required to evoke significant effects on skilled reaching and grasping^23^. We demonstrate that the means for this recovery is likely to be based upon the growth or sprouting of neurites through previously inhibitory substrates. Further, our study validates an in vitro and in vivo study where an autologous population of lentiviral mChABC transduced Schwann cells were implanted into the spinal cord following thoracic contusion injury^24^. Similar to our work, Kanno et al*.*^24^ demonstrated that mChABC removes CS-GAGs within the graft and surrounding host tissue and facilitates the integration of Schwann cells with host astrocytes. The degree and distance to which these cells intermingle is accurately reflected in our in vitro and in vivo findings. Indeed the echoing of the in vitro data with our in vivo observations and other studies suggests that, although our in vitro functional assays are simplistic models of the complex network, matrices, and cellular, chemical and electrical interactions which occur in the animal, they yield fundamental and translational information. Our data suggest that Schwann cells are able to integrate into this environment as they can myelinate neurites. However, a complete demonstration of integration in inhibitory environments would also illustrate local trophic support and formation of cellular scaffolds produced by the cells. Nevertheless, here we provide evidence of a cellular mechanism governing the intermingling of cells at inhibitory boundaries caused by the changes in cellular migration and adhesion of the Schwann cells. Further, we have identified a mechanism through which Schwann cell migration, adhesion and intermingling can be affected by ChABC. However, there are many additional processes that could be affected by use of the enzyme, for example, effects on all ECM components, signalling pathways, ligands, receptor binding and autophagy^2,64,70^. These need to be addressed to determine all the mechanisms through which mChABC may mediate effects at the CNS/PNS boundary.

bChABC has been used to great effect in combination with peripheral nerve^71–74^ and Schwann cell^61,75^ grafts to yield functional recovery following severe SCIs. However, for the first time we demonstrate that secretion of mChABC is more effective than a single application of bChABC at producing these effects. These data have substantial weight in the development of genetic modification through mChABC as a treatment for SCI. However, a full biochemical analysis of mChABC activity, as compared to the commercially available bChABC, is still required.

Accounting for number of cells and vector particles used, the transduced Schwann cells in this study produce similar yields of active mChABC to that shown in vivo^24^, demonstrating the robust application of this genetic modification treatment. Moreover, we uniquely demonstrate that these cell populations produce constant amounts of enzyme over time. Single aliquots of enzyme have also been shown to retain activity for longer at 37 °C than bChABC, suggesting stability. These are similar data to forms of the enzyme stabilised by trehalose^17^. This modified stability at biological temperatures means that any amount of enzyme produced from transduced cells would remain active for longer, increasing applicability of the treatment approach. This is important as in vivo studies suggest that, while the functional and anatomical effects of the enzyme are present at 8–12 weeks, lentiviral mChABC has reduced cellular production 3 months following transduction^21,24^. These data suggest that purified conditioned medium from mChABC transduced cells might be a viable alternative to treatment with commercially available purified bChABC.

The action of bChABC and mChABC within this study has revealed further insight into the mechanisms of CSPG mediated inhibition of cellular migration and growth. While known to exert cellular effects due to their strong negative charge^76^, CSPGs have been shown to alter function through receptor binding^77–80^. CSPGs also mediate effects through integrin-dependent mechanisms^31,41,44,51^. Here we have shown that when grown on an aggrecan substrate, Schwann cell phospho-FAK is reduced, while total FAK levels within the cells remains constant, suggesting a reduction in intracellular integrin signalling. Interestingly, we further demonstrate that the action of mChABC and bChABC can rescue this effect suggesting that it is the aggrecan GAG chains, and not the core protein, that interferes with integrin signalling. This demonstrates that, despite genetic modification, both mChABC and bChABC operate through similar mechanistic pathways. Utilising this in vitro model, determining the complete downstream intracellular pathways affected by CSPG binding on cytoskeletal dynamics and how they are affected by mChABC activity would provide significant insight.

Our data support the continued development of second-generation ChABC enzymes for the treatment of SCI. There have been a number of viral chondroitinase AC and ABC vectors developed^81–83^. However, we have shown that mChABC uniquely demonstrates a high and robust yield and substantial functional effects in vitro and in vivo. However, for additional effects on neuronal growth our study suggests that mChABC should be combined with treatments known to modulate Schwann cell and axonal behaviour such as integrins, N-cadherins, and Ephs^31,42,84,85^. The effectiveness of lentiviral mChABC treatment both in vitro and in vivo shows the significance of modifying the ECM in the treatment of SCI. Here we have explained a cellular mechanism underlying the functional and anatomical recovery shown in mChABC use. However, to further develop this as a clinical treatment, the construct should be optimised, perhaps by placing it in an increasingly stable vector such as an adeno-associated virus^86^, or assessing the risk of insertional mutagenesis in host cells^87^.

In summary, we provide evidence demonstrating the potent effects that lentiviral mChABC has upon glia cell dynamics and behaviour, facilitating cellular intermingling and neurite growth. These data highlight the importance of matrix modification in the treatment and recovery following SCI and provides a cellular mechanism for mChABC induced functional and behavioural recovery shown in in vivo studies. Furthermore, we demonstrate that this modified enzyme is stably secreted in high yield with functional activity similar to, or greater than, the commercially available bChABC. These findings have significant implications for the continued development of second-generation enzymes for the treatment of SCI and other CNS diseases.

## Methods

### Antibodies and reagents

Antibodies used are detailed in Tables 1 and 2. Dulbecco’s modified Eagle’s medium (DMEM) with and without phenol red, Hanks’ balanced salt solution (HBSS), sodium pyruvate, and foetal calf serum (FCS) were purchased from Thermo Fisher Scientific. Penicillin–streptomycin-fungizone (PSF, Sigma), nerve growth factor (NGF, Sigma), ITS + (insulin–transferrin–sodium selenite with bovine serum albumin (BSA) and linoleic acid; BD Bioscience), aphidicolin mitotic inhibitor, poly-D-lysine (PDL), laminin, collagenase and trypsin (Sigma) were used at concentrations specified in the text. The aggrecan used (A1960; Sigma) had a molecular weight > 2,500 kDa and with approximately 100–150 glycosaminoglycan chains attached. Bacterial chondroitinase ABC (Sigma) was used in a buffer containing 0.1 M sodium acetate apart from biochemical characterisation where the buffer comprised 50 mM Trizma base and 50 mM sodium acetate (pH 8.0; Sigma). Cell counts were conducted using a Countess automated cell counter (Thermo Fisher Scientific). The lenti-viral (LV) constructs LV-mChABC (5.6 × 10^6^ TU/μL) and LV-fGFP (farnesylated green fluorescent protein; 3.4 × 10^8^ TU/μL) were constructed under a cytomegalovirus (CMV) promoter. Lenti-viral vectors were produced according to standard protocols^88^.

**Table 1:** List of primary antibodies. (GFAP) Glial fibrillary acidic protein, (GFP) green fluorescent protein, (pFAK) phosphorylated FAK, (MBP) myelin basic protein, (ICC) immunocytochemistry, and (WB) western blot.

Antibody	Clonality	Isotype	Host	Concentration	Company
1B5	IgG	Monoclonal	Mouse	1:500 (ICC) 1:1,000 (WB)	Seikagaku
CS-56	IgM	Monoclonal	Mouse	1:300 (ICC) 1:1,000 (WB)	Sigma
Laminin	IgG	Polyclonal	Rabbit	1:300 (ICC)	Sigma
βIII-tubulin	IgG	Polyclonal	Rabbit	1:2000 (ICC)	Covance
βIII-tubulin	IgG	Monoclonal	Mouse	1:200	ThermoFisher Scientific
pFAK (try397)	IgG	Polyclonal	Rabbit	1:100 (ICC)	Invitrogen
FAK	IgG	Monoclonal	Mouse	1:100 (ICC)	Millipore
GFP	IgG	Polyclonal	Rabbit	1:1,000 (ICC)	AbCam
GFP	IgG	Polyclonal	Rabbit	1:1,000 (ICC)	Life Technologies
GFAP	IgG	Polyclonal	Rabbit	1:500 (ICC)	DAKO
GFAP	IgG	Monoclonal	Mouse	1:300 (ICC)	Sigma
GFAP	IgG	Monoclonal	Chicken	1:2000 (ICC)	AbCam
p75^NTR^	IgG	Monoclonal	Human	1:50 (ICC)	Millipore
p75^NTR^	IgG	Monoclonal	Mouse	1:1,000 (ICC)	Millipore
Aggrecan (Cat 301)	IgG	Monoclonal	Mouse	1:300 (ICC) 1:500 (WB)	Millipore
Aggrecan (AB1031)	IgG	Polyclonal	Rabbit	1:300 (ICC) 1:500 (WB)	Millipore
Ki67 (PP-67)	IgG	Monoclonal	Mouse	1:200 (ICC)	AbCam
MBP	IgG	Polyclonal	Rabbit	1:1,000 (ICC)	AbCam
MBP (G7G)	IgG	Monoclonal	Mouse	1:2000 (ICC)	AbCam
S100	IgG	Monoclonal	Mouse	1:100 (ICC)	ThermoFisher Scientific
Hoechst-33342				1:10,000 (ICC)	Sigma
Dappi (in ProLong Gold mounting medium)					ThermoFisher Scientific

**Table 2:** List of secondary antibodies. (ICC) immunocytochemistry, and (WB) western blot.

Host	Reactivity	Conjugate	Excitation wavelength (nm)	Emission wavelength (nm)	Concentration	Company
Donkey	Rabbit	Alexa fluor 488	495	519	1:500 (ICC)	Invitrogen
Donkey	Rabbit	Alexa fluor 568	578	603	1:500 (ICC)	Invitrogen
Donkey	Mouse	Alexa fluor 488	495	519	1:500 (ICC)	Invitrogen
Donkey	Mouse	Alexa fluor 568	578	603	1:500 (ICC)	Invitrogen
Donkey	Rabbit	Alexa fluor 488	495	519	1:500 (ICC)	ThermoFisher Scientific
Donkey	Mouse	Alexa fluor 594	579	750	1:500 (ICC)	ThermoFisher Scientific
Goat	Rabbit	Alexa fluor 488	495	519	1:500 (ICC)	Invitrogen
Goat	Rabbit	Alexa fluor 568	578	603	1:500 (ICC)	Invitrogen
Goat	Rabbit	Alexa fluor 660	663	690	1:500 (ICC)	Invitrogen
Goat	Mouse	Alexa fluor 488	495	519	1:500 (ICC)	Invitrogen
Goat	Mouse	Alexa fluor 568	578	603	1:500 (ICC)	Invitrogen
Goat	Mouse	Alexa fluor 660	663	690	1:500 (ICC)	Invitrogen
Goat	Mouse	Alexa fluor 568	578	603	1:500 (ICC)	Invitrogen
Goat	Chicken	Alexa fluor 488	495	519	1:500 (ICC)	Invitrogen
Goat	Mouse	Biotinylated			1:200 (ICC)	Vector
Goat	Mouse	HRP-conjugate			1:50,000 (WB)	Vector
	Streptavidin	Alexa fluor 568	578	603	1:500 (ICC)	Invitrogen

### Cell culture

Primary cells were isolated from postnatal day 1–3 (P1-3) Sprague Dawley rats. All experiments were approved by the University of Cambridge and were conducted in accordance with UK Animals (Scientific Procedures) Act 1986 (ASPA) under Home Office approval and licence. The health and welfare of all animals was monitored daily by veterinary staff and the study investigators at the University of Cambridge and was in accordance with the Animal Welfare Act 2006 and The Welfare of Farm Animals (England) Regulations 2007.

### Neonatal Schwann cells

Sciatic nerves were dissected, cleaned, and dissociated before trituration. The cells were suspended in supplemented DMEM (sDMEM; DMEM with 10% FCS, 2% PSF, bovine pituitary extract (BPE; Sigma, 10 μg/mL) and forskolin (Calbiochem, 2 μM)) then plated on PDL (25 μg/mL) and laminin (1 μg/mL) coated flasks. Cells were then treated with cytosine arabinoside (Ara-C; 1 × 10^5^ M; Sigma) and complement lysis (anti Thy1.1 antibody; 1:5 Hybridoma cell supernatant T11D7 and rabbit complement; 1:5; Serotec) before being maintained in sDMEM. For transduction, purified Schwann cells were seeded on PDL and laminin coated 6-well plates (1 × 10^5^ cells/well). Twelve hours later, LV-fGFP and/or LV-mChABC were added to the cells at MOIs of 2.5 and 10, respectively, in the presence of 0.625 μM polybrene (Sigma). Co-transductions were performed with the individual LV-fGFP and LV-mChABC vectors. The following day, the solution was washed off the cells.

### Neonatal astrocytes

The cerebral cortices were excised from neonatal rats, the meninges removed, the tissue diced and incubated with 0.1% trypsin for 30 min. The solution was triturated, centrifuged, the cells suspended in DMEM with 10% FCS, and plated on PDL coated flasks. Contaminating oligodendrocyte precursor cells and microglia were removed by shaking the culture overnight at 500 rpm in a 37 °C incubator shaker (Luckham R300). The astrocytes were maintained in DMEM supplemented with 10% FCS and 2% PSF. Astrocytes displayed a mature/reactive phenotype demonstrated through GFAP staining and production of laminin and CSPGs demonstrated through CS56 and aggrecan staining (Fig. 3).

### Neonatal dorsal root ganglion (DRG) neurons

The 30 neonatal DRG pairs were extracted and dissociated. The suspension was centrifuged through a 15% BSA density gradient at 100 × g and the cells suspended in DMEM supplemented with ITS + (1:100), PSF (1:100) and the growth supplement NGF (10 ng/mL). Cells were plated on to PDL and laminin (1 μg/mL) coated flasks and incubated at 37 °C with 7% CO_2_.

### Functional assay design and quantification

#### Schwann cell inverted coverslip migration assay

Schwann cells (1 × 10^6^ cells/mL) were plated on PDL and laminin coated coverslip fragments (~ 1 mm^2^) in a 20 μL droplet and incubated until confluent. Test substrates were coated with PDL and either aggrecan (20 μg/mL), laminin (1 μg/mL), or laminin and aggrecan. Coverslip fragments (with cells) were inverted on the test substrates, and cells were allowed to migrate for 24 h in DMEM with 10% FCS before live imaging, analysis, and fixation in 4% paraformaldehyde (PFA). When required, the ECM or astrocyte monolayer was treated with 0.2 U/mL bChABC for 1 h at 37 °C. In assays requiring a mitotic inhibitor, aphidicolin (1 μg/mL; or equivalent volume of DMSO) was supplemented to the sDMEM during migration. When migrating Schwann cells upon a confluent monolayer of astrocytes, the PNS glia were labelled with vibrant DiI (Thermo Fisher Scientific). The confluent astrocyte monolayer was confirmed through microscopy and coverslip fragments only plated on areas know to be confluent. All data was normalised to control conditions (naïve Schwann cells migrated upon a PDL). Schwann cell migration was assessed live under phase contrast (10 × magnification, Nikon) or fluorescent microscope (10 × magnification, Leica). Quantification using an eyepiece grid reticule (Nikon) included: the maximum distance of cell body migration from the edge of a coverslip fragment (assessed at 90° to the edge of the coverslip), and the number of cells migrating at various distances (assessed in 100μm^2^ bins; Supplementary Fig. 5a).

#### Preparation of astrocytic monolayers and matrix

Purified astrocytes (2 × 10^5^ cells/mL) were plated on PDL coated coverslips and left for 48 h to form dense monolayers. For immunocytochemistry, medium was replaced with that collected and concentrated from transduced primary Schwann cells or cDMEM supplemented with 0.2 U/mL bChABC for 1 h at 37 °C, before fixation with 4% PFA. For the preparation of astrocytic matrix, the confluent astrocyte monolayers were lysed by hypotonicity through 45–50 min exposure to sterile water. The matrices were washed 3 times to remove debris and stored at 4 °C in PBS for maximum of 24 h until use.

#### Confrontation assay

Sterile silicone gaskets with 9 mm^2^ wells (Sigma) were placed on Permanox™ slides (Nunc, LabTEK). 2-hole silicone cell culture inserts (Ibidi) were placed into the PDL coated wells. Purified primary Schwann cells (7 × 10^5^ cells/mL) and astrocytes (4.6 × 10^5^ cells/mL) were each plated into one of the insert holes, 0.5 mm apart. After 12 h, the insert was removed and cells washed. Cultures were grown for 5 days in sDMEM until a clear boundary was established. If required, bChABC (100 mU) was placed in the cell medium for 1 h at 37 °C daily. For experiments assessing neurite outgrowth, dissociated DRGs (1 × 10^4^cells/mL) were plated over the boundary. Cells were grown at 37° for 2 days in DMEM supplemented with 2% PSF, 10% FCS, BPE (10 μg/mL), and NGF (10 ng/mL) with medium changed every 24 h. All experiments were terminated through cell fixation with 4% PFA. All data was normalised to control conditions (naïve Schwann cells and astrocytes). Experiments were analysed using fluorescent microscopy (Leica6000; following ICC) using Adobe Illustrator (CS5) via a methodology described previously^31^ (Supplementary Fig. 5b). The number of Schwann cells migrating into the glial cell population was counted at distances of 200, 400 and greater than 400 μm (63 × magnification). Neurite growth on Schwann cell-astrocyte confrontation assays was quantified using previously specified criteria^29^ (Supplementary Fig. 5c). A minimum of 10 neurites were counted per image.

#### Adhesion assay

Primary Schwann cells (2 × 10^5^ cells/mL) were plated on each coverslip and left to adhere at 37 °C for 1 h. The coverslips were then shaken with randomized rotation (100 rpm; Eppendorf) for 4 h at 37 °C, then washed to remove any non-attached cells. If required, 0.2U/mL bChABC was applied to the coverslip for 1 h at 37 °C. Assays conducted on pure substrate were analysed live using a phase contrast microscope (5 × magnification, Nikon) while those conducted on astrocyte monolayer were fixed with 4% PFA and then stained before fluorescent imaging (5 × magnification, Leica) and analysis. All data was normalised to control conditions (naïve Schwann cells adhering to PDL or astrocyte monolayer). Adhesion was assessed by scoring the number of cells in a defined 100 μm x 1000 μm area. A minimum of 15 images at random locations on each coverslip were taken at time points both before and after shaking.

#### Neurite outgrowth assay and assessment of myelination

Primary Schwann cells (1 × 10^5^ cells/mL) were plated on PDL and laminin coated coverslips incubated at 37 °C for 2 days in sDMEM until confluent. Dissociated DRGs (1 × 10^4^ cells/mL) were plated upon the Schwann cells and incubated at 37 °C for 2 days in DMEM supplemented with 2% PSF, 10% FCS, BPE (10 μg/mL), and NGF (10 ng/mL). Cells were washed and fixed with 4% PFA for ICC and analysis. All data was normalised to control conditions (DRG outgrowth on naïve Schwann cells). Neurite outgrowth was assessed following ICC with anti-β-III tubulin under fluorescent microscopy (Leica6000; 20 × magnification). Neurite myelination was assessed following ICC with anti-MBP, anti-β-III tubulin and anti-p75 under fluorescent microscopy (Leica6000; 20 × or 63 × magnification). A minimum of 20 randomly chosen neurites were imaged per coverslip.

### In vivo assessments

#### Animals

All animal surgeries were conducted in accordance with the United Kingdom Animals (Scientific Procedures) Act 1986. Adult female Sprague Dawley rats (200–220 g; Harlan Laboratories) were housed in groups of three or four, exposed to a normal 12-h dark–light cycle at 21 °C with free access to food, water and environmental enrichment ad libitum. The health and welfare of all animals was monitored daily by veterinary staff and the study investigators at Kings College London. Experimenters were blinded to treatment conditions during testing and histological assessment and sterile procedures were observed during all surgical techniques.

#### Contusion injury and intraspinal injections

Animals were anaesthetised with ketamine (60 mg/kg) and medetomidine (0.25 mg/ml). Upon reaching a surgical plane of anaesthesia, animals received a subcutaneous (s.c.) injection of Carprofen (5 mg/kg). Body temperature was maintained throughout the surgery at 37 ± 1 °C using a homothermic heat pad (Harvard Apparatus). Animals received a 150 kdyne midline contusion at T10 (Infinite Horizon impactor; Precision Systems Instrumentation). Following contusion, rats received intraspinal injections (2 × 0.5 μl; 1 mm rostral and caudal to the injury site) of protease-free bChABC (10 U/ml, n = 3, Seikagaku); LV-mChABC (n = 3) or LV-fGFP (n = 3). Animals were given s.c. injections of saline and recovered in a heated environment before transfer to their home cage. Analgesia and hydration were maintained for 5 days post-surgery in addition to nutritional support if the animal’s weight fell 5% below that determined pre-injury. No animal showed any adverse effects to the surgery or procedures performed.

#### Tissue processing

Two weeks following injury, animals were anesthetised (Euthatal, 80 mg/kg, i.p.) and transcardially perfused with 4% paraformaldehyde in 0.1 M phosphate buffer and the spinal cord removed. Following 2 h post-fixation, tissue was cryoprotected in 20% sucrose for 48 h. Following embedding in Optimum Cutting Temperature Compound, the spinal cord was cut into serial 20 μm sagittal sections.

### Immunocytochemistry

Cells/tissue were fixed for 10 min in 4% PFA, washed, and blocked in 0.3% Triton-X 100 (Sigma) and either 3% bovine serum albumin (BSA; Sigma) or 10% donkey serum (Stratech). Cells/tissue were incubated with primary antibody overnight at 4 °C and secondary antibody for 2 h at 25 °C. If required, Hoechst-33342 applied to the cells for 10 min at 25 °C. Coverslips were mounted onto slides with Fluorosave (Calbiochem) while tissue was mounted using ProLong Gold (ThermoFisher Scientific). For experiments with a biotinylated secondary, cells were additionally incubated in streptavidin for 2 h at 25 °C. Cells were analysed under fluorescent microscopy (Leica6000; 20, 40 and 63 × magnification). A minimum of 20 images were taken at random locations on each image. Images were analysed using ImageJ (NIH) software with the integrated pixel density assessed in a minimum of 10 areas positive for the epitope. Signal intensity readings were standardised for area and background. To determine the rate of cell division and transduction efficiency, the total number of cells was determined through Hoechst-33342 staining. Tissue was assessed under confocal microscopy (Zeiss; 20 × magnification).

### Cetylpyridinium chloride (CPC) turbidity assay

Cell culture medium (DMEM without phenol red, supplemented with sodium pyruvate and ITS +) was collected from naïve and transduced Schwann cells over 24–168 h at 37 °C. EDTA-free protease inhibitor cocktail (Roche) was added and the supernatant and concentrated using a 50 K centricon (Millipore). 5μL media samples were incubated with 50μL chondroitin sulphate A (CS-A; 20 μg; Sigma) at 37 °C for 30 min and denatured at 95 °C. 20μL of each sample was incubated with an equal volume of CPC reagent (containing 1:1 of 0.2% (w/v) CPC and 133 mM magnesium chloride; Fluka). Absorbance was measured at 405 nm using a μQuant™ Microplate Spectrophotometer (Biotek Instruments) and data adjusted for baseline based on the negative controls^89^.

### Reverse transcription-polymerase chain reaction (RT-PCR)

The following primers were used for RT-PCR: GFP: 5′-CCTGAAGTTCATCTGCACCAC-3′, 5′-TGCTCAGGTAGTGGTTGTCGG-3′; mChABC: 5′-GAAAATTTAGCGGCCATTGA-3′, 5′-TGATCAGATGTCTGCCTTGC-3′; HPRT: 5′-AGCTACTGTAATGATCAGTCAACG-3′, 5′-AGAGGTCCTTTTCACCAGCA-3′. Total RNA was extracted from naïve transduced primary Schwann cells using RiboPure (Ambion) and treated with DNase. Reverse transcription was performed using the Superscript II First-Strand Synthesis System (Theromo Fisher Scientific). PCR of the cDNA occurred using a MangoTaq Polymerase PCR Kit (Bioline) with an initial denaturation step at 94 °C for 3 min, followed by a specific number of cycles [GFP: 30; mChABC and hypoxanthine–guanine phophoribosyltransferase (HPRT): 38]. PCR products were analysed on a 1% agarose gel.

#### Western blotting

Cells were lysed with RIPA (radio-immunoprecipitation assay; Roche) supplemented with 5% protease inhibitor and phosphatase inhibitor cocktails (Roche). Protein concentration was determined using BCA Protein Assay Kit (Pierce). Protein extracts underwent SDS-PAGE and blotted onto a polyvinylidene difluoride (Hybond-P) membrane (GE Healthcare). Membranes were blocked in 5% skimmed milk in TBS-T before incubation with anti-1B5 antibody at 4 °C for 12 h. The membrane was then incubated in HRP-conjugated anti-mouse antibody at 25 °C for 2 h. Bands were visualized with ECL detecting reagents (GE Healthcare). Western blots were repeated a minimum of 3 times, using samples from independent batches.

#### Statistics

All experiments were assessed blind. A minimum of 5 repeats were conducted for each experiment with separate samples being collected from independent cell batches per condition. For cell culture assays, each treatment condition was repeated a minimum of 3 times within each experiment. The parameters were compared between control and the test group using the one-way ANOVA with post-hoc Bonferroni. Significance values are represented as * = *p* < 0.05, ** = *p* < 0.01, *** = *p* < 0.001, graphs show means ± SEM.

## Supplementary information

Supplementary file1 (PDF 932 kb)

## Data Availability

Correspondence and requests for material should be addressed to philippa.warren@kcl.ac.uk. The data sets generated and/or analysed during the current study are available from the corresponding author on reasonable request. Reprints and permissions information is available at www.nature.com/reprints.
