# Supplementary material for: Secretion of a mammalian chondroitinase ABC aids glial integration at PNS/CNS boundaries
Source: Sci Rep. 2020 Jul 9;10:11262. doi: 10.1038/s41598-020-67526-0 (PMC7347606; doi:10.1038/s41598-020-67526-0)
Supplement: Supplementary file 1 — Supplementary file1 (PDF 932 kb) [file 41598_2020_67526_MOESM1_ESM.pdf]

# Title: Secretion of a mammalian chondroitinase ABC aids glial integration at PNS/CNS boundaries

**Authors:** Philippa M. Warren, Melissa R. Andrews, Marc Smith, Katalin Bartus, Elizabeth J. Bradbury, Joost Verhaagen, James W. Fawcett, Jessica C. F. Kwok

**Supplementary Figures 1-5**

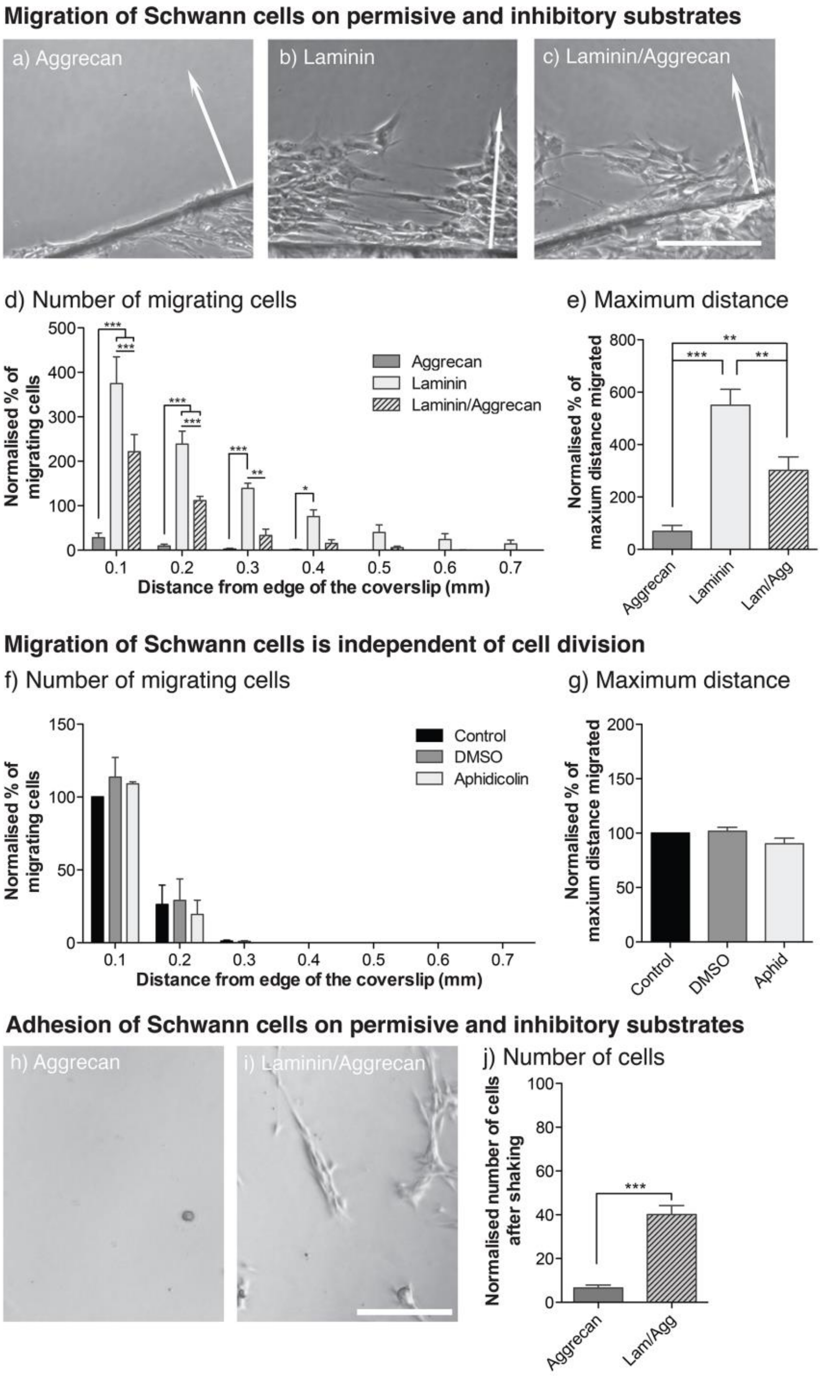


**Supplementary Figure 1:** Schwann cell migration and adhesion is substrate dependent. a-e) Schwann cell migration on a) aggrecan, b) laminin, and c) laminin and aggrecan. Scale bar = 100μm. Arrows indicate direction of migration. d) Number of Schwann cells migrating is substrate dependent (N=4, two-way ANOVA: substrate F(2,105)=113.93, *p*<0.0001, distance F(6,105)=67.6, *p*<0.0001). e) Maximum distance of Schwann cell migration alters in a substrate dependent manner (N=4, one-way ANOVA: F(2,15)=35.13, *p*<0.0001). f) Number of Schwann cell migrating is independent of cell division (N=5, two-way ANOVA: substrate F(2,42)=0.37, *p*=0.6904, distance F(6,42)=150.29, *p*<0.0001). g) Maximum distance of Schwann cell migration is independent of cell division (N=5, one-way ANOVA: F(4,10)=110.6, *p*<0.0001). h-j) Schwann cell adhesion on h) aggrecan, i) laminin and aggrecan. Scale bar = 100μm. j) Schwann cells adhere less to aggrecan coated coverslip (N=3, T-test: F(3,3)=9.139, *p*=0.0001).

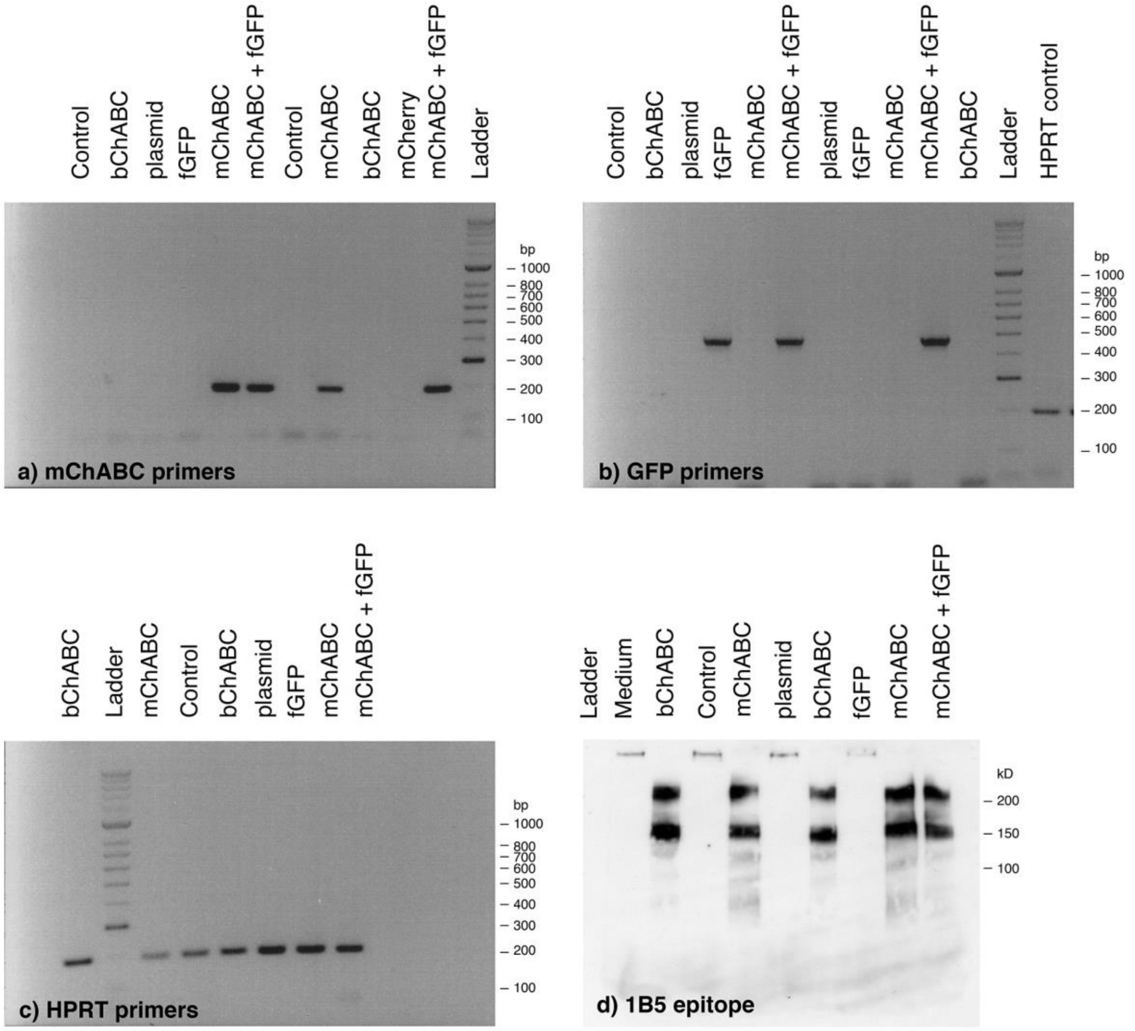


**Supplementary Figure 2:** Full-length gels and blots of the cropped images taken from Figure 1 e-f). a-c) RT-PCR of cells with HPRT, mChABC and GFP primers. All images taken in the same field and exposure. d) Western blot of cell medium probed using anti-1B5 antibody. DNA and proteins were quantified to ensure equal gel loading.

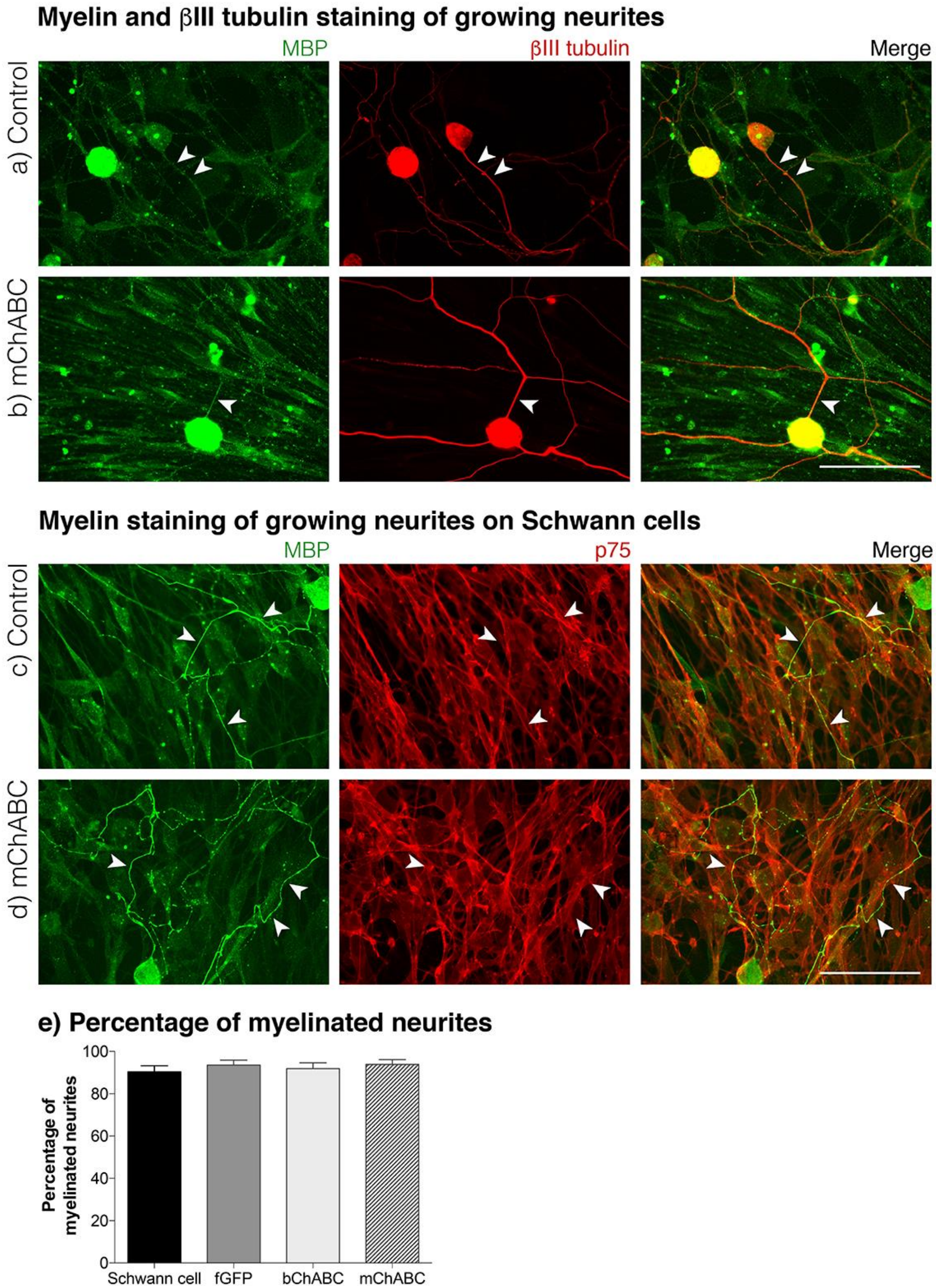


**Supplementary Figure 3:** mChABC secretion from Schwann cells does not affect myelination of DRG neurites. a-b) Images show myelin basic protein (anti-MBP; green) secreted from Schwann cells and DRG neurites (anti-βIII-tubulin; red) for a) control and b) mChABC transduced cells. Arrows indicate myelinated DRGs not growing along Schwann cells. c-d) Images show myelin basic protein (anti-MBP; green) secreted from Schwann cells (p75; red) for c) control and d) mChABC transduced cells. Arrows indicate MBP protein that is not co-localised with Schwann cells. e) Secretion of mChABC from Schwann cells does not affect neurite myelination (N=3, one-way ANOVA: $F(3,119)=0.388$, $p$=ns). For all panels, scale bar = 100μm.

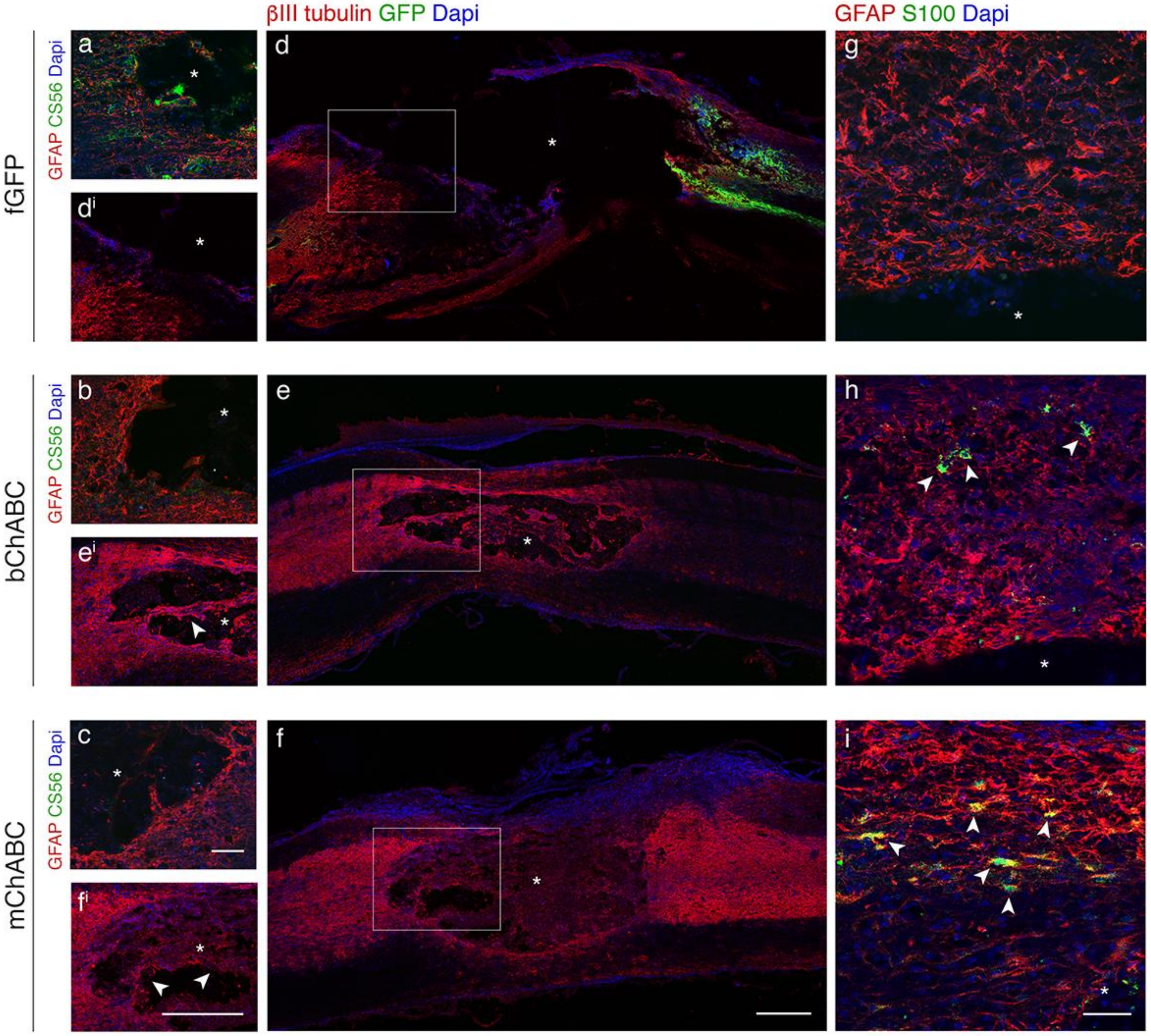


**Supplementary Figure 4:** mChABC facilitates neuroprotection and Schwann cell intermingling following spinal contusion. a-c) mChABC and bChABC show reductions in chondroitin sulphate (anti-CS56; green) compared to control demonstrating activity of the enzyme while showing similar reactive astrocyte (anti-GFAP; red) staining. Scale bar = 200μm. d-f) mChABC shows more neuroprotection and growth of neurons (anti-βIII-tubulin; red) through the lesion then bChABC and fGFP (anti-GFP; green) infused tissue. Arrows demonstrate continued presence of neurons through the lesion site. $X^{i}$ images show higher resolution of the lesion area indicated in the main figure. Scale bar = 500μm. g-i) mChABC secretion from cells increases the number of Schwann cells (anti-S100; green) intermingling within the reactive astrocyte (anti-GFAP; red) populations following injury. Arrows show the presence of Schwann cells. Scale bar = 50μm. All panels show sagittal sections with Dapi (blue), where * represent the lesion.

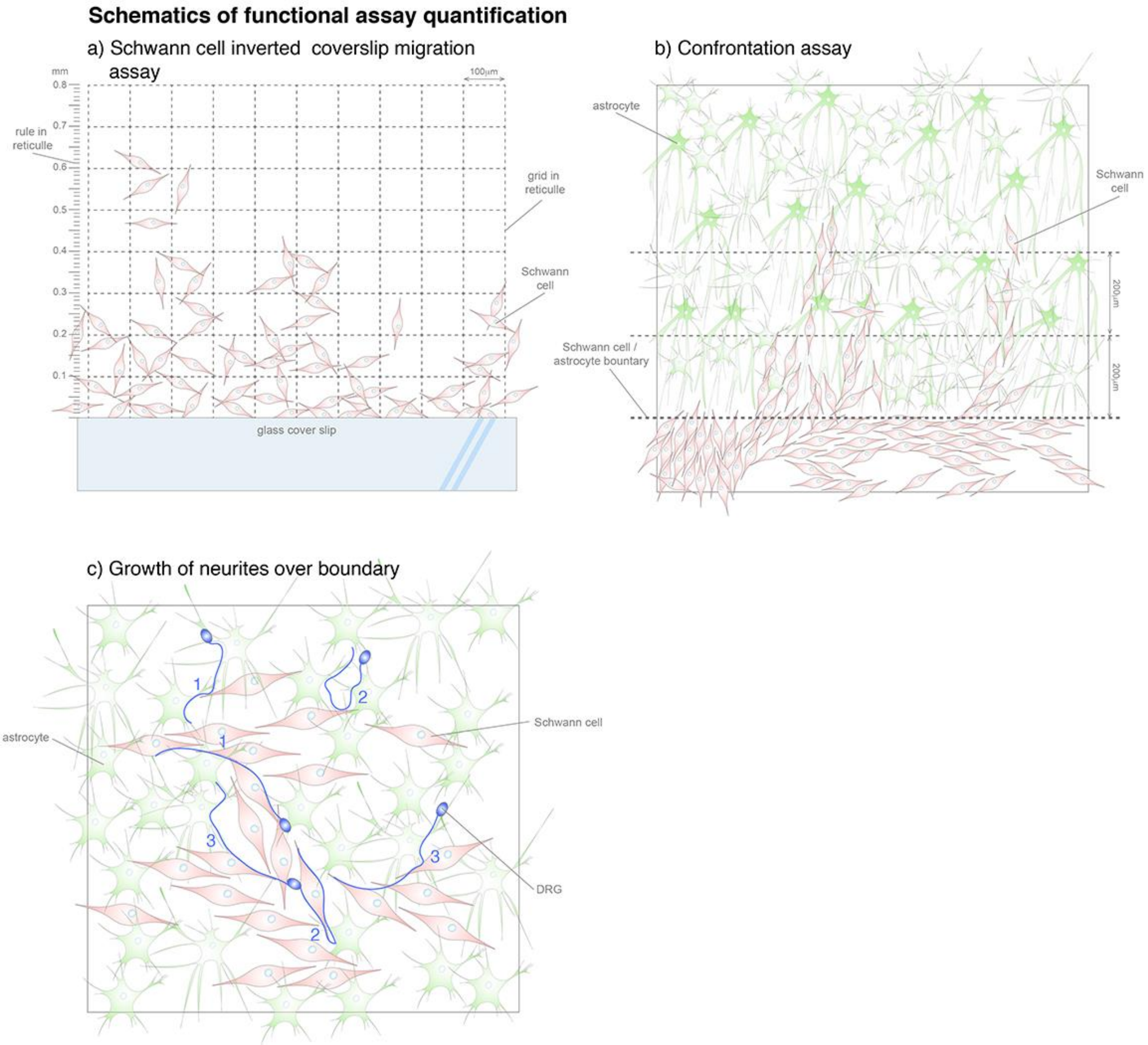


**Supplementary Figure 5:** Schematics for the assessment of cell culture assays. a) Quantification of the Schwann cell inverted coverslip migration assay involved counting cell bodies in 100μm$^2$ bins from the edge of the coverslip using a microscope eyepiece grid reticule (Nikon). The maximum distance of cell body migration from the edge of a coverslip fragment (assessed at 90° to the edge of the coverslip using rule in graticule), and the number of cells migrating at various distances was assessed. b) Quantification of the confrontation assay occurred from ICC images using Adobe Illustrator (CS5). The Schwann cell/astrocyte boundary was identified. The number of cells migrating into the astrocyte population was counted in three 200μm bins from the boundary edge using the cell bodies. c) Illustration of axon behaviour over the Schwann cell/astrocyte boundary: 1 = neurites stay growing on cell type of origin when presented with an alternative; 2 = neurites cross over to another cell type but immediately cross back to cell type of origin; 3 = neurites cross over the cellular boundary continue growing on the second cell type.
